# A Study of Heredity Insanity in the Light of the Mendelian Theory

**Published:** 1912-08

**Authors:** A. J. Rosanoff, Florence I. Orr

**Affiliations:** Kings Paik State Hospital, Kings Park, N. Y.; Kings Paik State Hospital, Kings Park, N. Y.


### A Study of Heredity Insanity in the Light of the Mendelian Theory

By A. J. ROSANOFF, M.D., and FLORENCE I. ORR, B.S.
Kings Paık State Hospital, Kings Park, N. Y.
From *American Journal of Insanity*, October, 1911

The authors have taken up the present study with a view to determine whether a neuropathic constitution is transmitted, in cases of insanity, in the manner of a Mendelian trait, and they state briefly the Mendelian theory as follows:

"The total inheritance of an individual is divisible into unit characters, each of which is, as a general rule, inherited independently of all other characters and may therefore be studied without reference to them. The inheritance of any such character is believed to be dependent upon the presence in the germ plasm of a unit of substance called a *determiner*."

A character may be dominant or recessive and these characters are represented in the formulae by the symbols D and R respectively.

The pedigrees of 72 families have been arranged in a table showing the types of matings, and the actual findings compared to that which would be theoretically expected, and the findings are surprisingly similar. Thus, out of a total of 206 matings of all types in these 72 families there were 1097 offspring. The neuropathic offspring showed an actual finding of 351 as against the theoretical expectation of 359, and the normal offspring gave 586 as against a theoretical expectation of 578, and they regard this as definitely establishing the fact that the neuropathic constitution is transmitted as a recessive trait, in accordance with the Mendelian theory.

Various neuropathic equivalents are found in the ancestry of the insane as well as in the families of epileptics. In the families of patients suffering from manic-depressive insanity they find not only subjects clearly recognized as insane, but also others described as high-strung, excitable, abnormally selfish, awful temper, periodic drinker, severe blue spells, etc. In the pedigrees of dementia præcox they find relatives described as cranky, stubborn, queer, has phobias, suspicious of friends and relatives, etc.

The neuropathic equivalents of epilepsy include hemicrania, recurrent sick headaches, fainting spells, nervous figety make-up, etc.

All these conditions are graphically shown in the 73 pedigree charts which accompany the article.

The following conclusions are drawn:—

1. The neuropathic constitution is transmitted from generation to generation in the manner of a trait which is, in the Mendelain sense, recessive to the normal condition. Rules of theoretical expectation are accordingly as follows:

a. Both parents being neuropathic, all children will be neuropathic.

b. One parent being normal, but with neuropathic taint from one grandparent, and the other parent being neuropathic, half the children will be neuropathic and half will be normal but capable of transmitting the neuropathic make-up to their progeny.

c. One parent being normal and of pure normal ancestry and the other parent being neuropathic, all the children will be normal but capable of transmitting the neuropathic make-up to their progeny.

d. Both parents being normal, but each with the neuropathic taint from one grandparent, one-fourth the children will be normal and not capable of transmitting the neuropathic make-up to their progeny,one-half will be normal but capable of transmitting the neuropathic make-up, and the remaining one-fourth will be neuropathic.

e. Both parents being normal, one of pure normal ancestry and the other with the neuropathic taint from one grandparent, all the children will be normal, half of them will be acpable, and half not capable of transmitting the neuropathic make-up to their progeny.

f. Both parents being normal and of pure normal ancestry, all the children will be normal and not capable of transmitting the neuropathic make-up to their progeny.

